# Acellular bioscaffolds redirect cardiac fibroblasts and promote functional tissue repair in rodents and humans with myocardial injury

**DOI:** 10.1038/s41598-020-66327-9

**Published:** 2020-06-11

**Authors:** Daniyil A. Svystonyuk, Holly E. M. Mewhort, Ali Fatehi Hassanabad, Bobak Heydari, Yoko Mikami, Jeannine D. Turnbull, Guoqi Teng, Darrell D. Belke, Karl T. Wagner, Samar A. Tarraf, Elena S. DiMartino, James A. White, Jacqueline A. Flewitt, Matthew Cheung, David G. Guzzardi, Sean Kang, Paul W. M. Fedak

**Affiliations:** 1 Section of Cardiac Surgery, Department of Cardiac Sciences, Cumming School of Medicine, Libin Cardiovascular Institute, University of Calgary, Calgary, Alberta Canada; 2 Department of Radiology, Cumming School of Medicine, Stephenson Cardiac Imaging Centre, Libin Cardiovascular Institute, University of Calgary, Calgary, Alberta Canada; 3 Department of Civil Engineering, Libin Cardiovascular Institute and Centre for Bioengineering Research and Education, University of Calgary, Calgary, Alberta Canada

**Keywords:** Tissue engineering, Cardiac regeneration, Heart failure, Translational research

## Abstract

Coronary heart disease is a leading cause of death. Tissue remodeling and fibrosis results in cardiac pump dysfunction and ischemic heart failure. Cardiac fibroblasts may rebuild damaged tissues when prompted by suitable environmental cues. Here, we use acellular biologic extracellular matrix scaffolds (bioscaffolds) to stimulate pathways of muscle repair and restore tissue function. We show that acellular bioscaffolds with bioinductive properties can redirect cardiac fibroblasts to rebuild microvascular networks and avoid tissue fibrosis. Specifically, when human cardiac fibroblasts are combined with bioactive scaffolds, gene expression is upregulated and paracrine mediators are released that promote vasculogenesis and prevent scarring. We assess these properties in rodents with myocardial infarction and observe bioscaffolds to redirect fibroblasts, reduce tissue fibrosis and prevent maladaptive structural remodeling. Our preclinical data confirms that acellular bioscaffold therapy provides an appropriate microenvironment to stimulate pathways of functional repair. We translate our observations to patients with coronary heart disease by conducting a first-in-human observational cohort study. We show that bioscaffold therapy is associated with improved perfusion of infarcted myocardium, reduced myocardial scar burden, and reverse structural remodeling. We establish that clinical use of acellular bioscaffolds is feasible and offers a new frontier to enhance surgical revascularization of ischemic heart muscle.

## Introduction

Coronary heart disease with ischemic myocardial injury and progression to heart failure is a leading cause of death worldwide^1^. Contemporary medical and interventional therapies can restore coronary artery inflow, alleviate symptoms, and improve survival. However, restoring the function of myocardial tissues damaged by ischemic injury is challenging. Loss of cardiac performance is strongly predictive of a poor prognosis^2^.

Tissue repair after injury is a highly regulated but imperfect process. In response to injury, heart muscle heals primarily by progressive fibrosis that can impair tissue function. Heart muscle injury is characterized by recruitment, proliferation and activation of cardiac fibroblasts. Cardiac fibroblasts produce extracellular matrix (ECM) components and heal injuries by creating fibrotic scars^3^. Infarct scarring is an adaptive mechanism that serves to preserve mechanical tissue integrity after the threat of injury. However, fibroblast cell actions can persist long after the scar is healed. Persistent pro-fibrotic cell activity is maladaptive and results in tissue fibrosis, structural chamber remodeling, and reduced cardiac pump function that can lead to clinical decompensation as end-stage heart failure^4^.

Heart scars have a dynamic microenvironment with heterogeneous populations of cardiac fibroblasts^5^. With appropriate environmental cues, fibroblasts can help coordinate cardiac repair and functional recovery^6^. For example, a subset of cardiac fibroblasts was recently shown to help rebuild microvascular networks following ischemic injury^7^. Fibroblast responses are highly contextual and their phenotypic fate is determined by signals emanating from the local microenvironment^8,9^. Accordingly, efforts to modify and control fibroblasts within healing tissues may result in an effective therapeutic strategy to reduce fibrotic healing and restore muscle function.

Acellular biologic ECM scaffolds can activate endogenous tissue repair processes^10^. Bioscaffolds are a reservoir for growth factors, matricellular proteins and bioactive vesicles^11–13^. We propose that acellular bioscaffolds can be leveraged as a directed signalling niche capable of targeting and redirecting fibroblast activities. Redirecting cardiac fibroblasts toward adaptive repair may help reduce tissue fibrosis and restore microvascular networks within ischemic muscle and in so doing, promote functional recovery.

In this study, we investigate the translational potential of acellular bioscaffolds for cardiac repair. We compare acellular scaffolds with bioactive properties intact against bioscaffolds with experimentally reduced bioinductive constituents. We show that human cardiac fibroblasts have attenuated pro-fibrotic activity when combined with bioactive scaffolds. The bioscaffold directs fibroblasts to secrete pro-vasculogenic mediators that can promote new blood vessel assembly. Using a rodent model of human myocardial infarction (MI) we compare the effects of acellular bioscaffolds on fibroblast-mediated pathways of cardiac remodeling. We show reduced interstitial fibrosis, wall thinning, and LV dilatation with bioscaffold therapy in rodents. We translate our preclinical observations to humans with ischemic injury. We illustrate that human bioscaffold therapy during surgical revascularization is feasible and observed effects are consistent with cell-based bioassays and animal models of human disease.

## Results

### Bioscaffolds downregulate fibrotic genes and upregulate vasculogenic genes

To determine the effects of bioscaffolds on human cardiac fibroblast phenotypes we used RNA-sequencing analysis to compare the gene expression profiles of human cardiac fibroblasts on intact bioscaffolds (with preserved bioactive constituents) with neutralized scaffolds (which were chemically treated to remove ECM-adsorbed bioactive constituents) (Supplementary Fig. 1A). RNA sequencing analysis revealed 650 differentially regulated genes between human cardiac fibroblasts seeded on intact versus neutralized scaffolds. Relevant genes are summarized in Supplementary Table 4. There were no statistically significant changes in gene expression in cells seeded on tissue culture plastic versus neutralized scaffolds.

**Figure 1:**
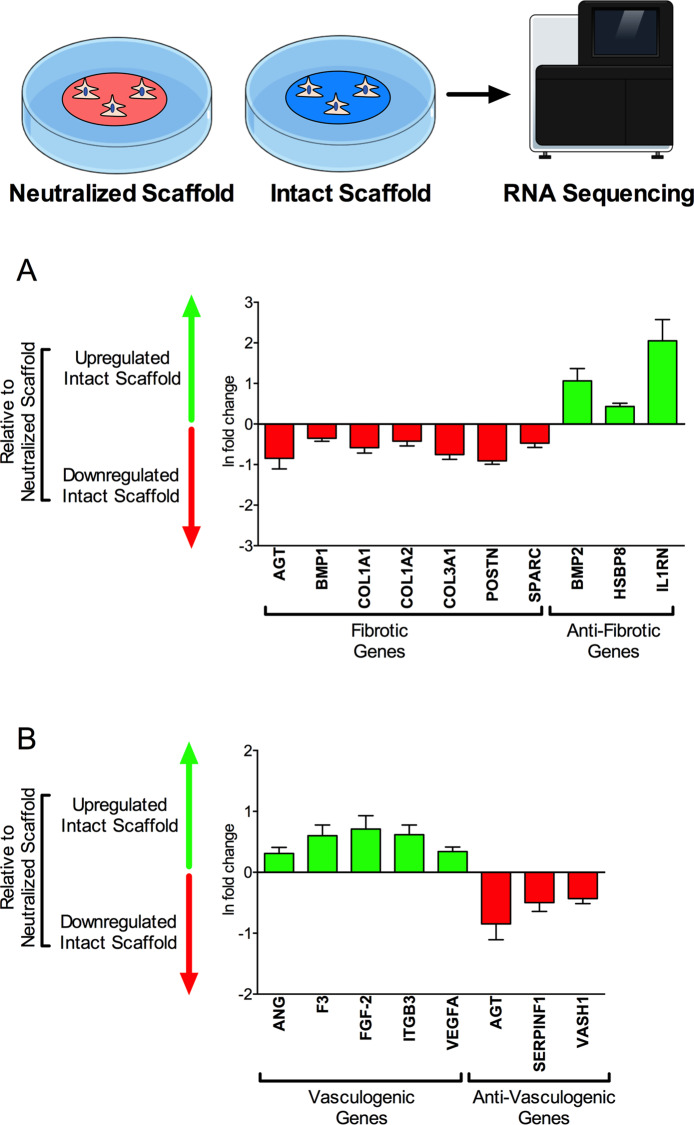
RNA Sequencing of Human Cardiac Fibroblasts on Bioscaffolds. (**A**) Selected genes associated with cardiac fibrosis are downregulated in human cardiac fibroblasts on intact bioscaffolds relative to matched cells on neutralized bioscaffolds (N = 4 subjects/group). (**B**) Genes associated with blood vessel assembly are upregulated in human cardiac fibroblasts on intact bioscaffolds relative to matched cells on neutralized scaffolds (N = 4 subjects/group). Values reported are group mean ± SD. All represented data had false discovery rate corrected-P values <0.05 as determined using the Benjamin-Hochberg procedure for multiple comparisons. Illustrations were prepared using MindtheGraph software (www.mindthegraph.com) and used under the creative commons license (https://creativecommons.org/licenses/by-sa/4.0/deed.en).

Human cardiac fibroblasts on intact bioscaffolds downregulated selected genes associated with cardiac fibrosis relative to matched cells on neutralized ECM scaffolds. Specific genes with differential expression included angiotensinogen (*AGT*; −0.85 ± 0.26 ln fold-change), bone morphogenetic protein-1 (*BMP-1*; −0.35 ± 0.07 ln fold-change), collagen type-1-alpha-1 (*COL1A1*; −0.58 ± 0.13 ln fold-change), collagen type-1-alpha-2 (*COL1A2*; −0.42 ± 0.12 ln fold-change), collagen type-3-alpha-1 (*COL3A1*; −0.76 ± 0.12 ln fold-change), periostin (*POSTN*; −0.91 ± 0.09 ln fold-change), and secreted protein acidic and rich in cysteine (*SPARC*; −0.47 ± 0.11 ln fold-change). Conversely, gene expression of anti-fibrotic proteins was upregulated in human cardiac fibroblasts on intact bioscaffolds relative to neutralized scaffolds. Specific genes included bone morphogenetic protein-2 (*BMP2*; 1.06 ± 0.31 ln fold-change), heat shock binding protein 8 (*HSBP8*; 0.43 ± 0.08 ln fold-change), and interleukin-1 receptor antagonist (*ILRN*; 2.05 ± 0.52 ln fold-change)(Fig. 1A).

Genes favoring blood vessel assembly were upregulated in human cardiac fibroblasts on intact bioscaffolds relative to cells on neutralized scaffolds. Increased expression was observed for angiogenin (*ANG*; 0.31 ± 0.10 ln fold-change), tissue factor-3 (*F3*; 0.60 ± 0.18 ln fold-change), basic fibroblast growth factor (*FGF2*; 0.71 ± 0.22 ln fold-change), integrin beta-3 (*ITGB3*; 0.62 ± 0.16 ln fold-change), and vascular endothelial growth factor (*VEGFA*; 0.34 ± 0.07 ln fold-change). Conversely, human cardiac fibroblasts on intact bioscaffolds show downregulation of gene expression for proteins associated with inhibition of vasculogenesis. These genes included angiotensinogen (*AGT*; −0.85 ± 0.26 ln fold-change), pigment epithelium-derived factor (*SERPINF1*; −0.50 ± 0.14 ln fold-change), and vasohibin (*VASH1*; −0.43 ± 0.08 ln fold-change) (Fig. 1B). Accepting that biophysical properties of tissues can modify fibroblast phenotypes, we confirmed that chemical treatments of the bioscaffolds did not change the mechanical properties of the biomaterial (Supplementary Fig. 1B). Recognizing that cell health can affect gene expression, we establish that fibroblast viability is not decreased by chemical neutralization of the bioscaffold (Supplementary Fig. 2). These data illustrate that human cardiac fibroblasts can be redirected toward a less fibrotic and pro-reparative vasculogenic phenotype by interaction with bioactive scaffolds.

### Human cardiac fibroblast fibrotic activity is attenuated by bioscaffolds

**Figure 2:**
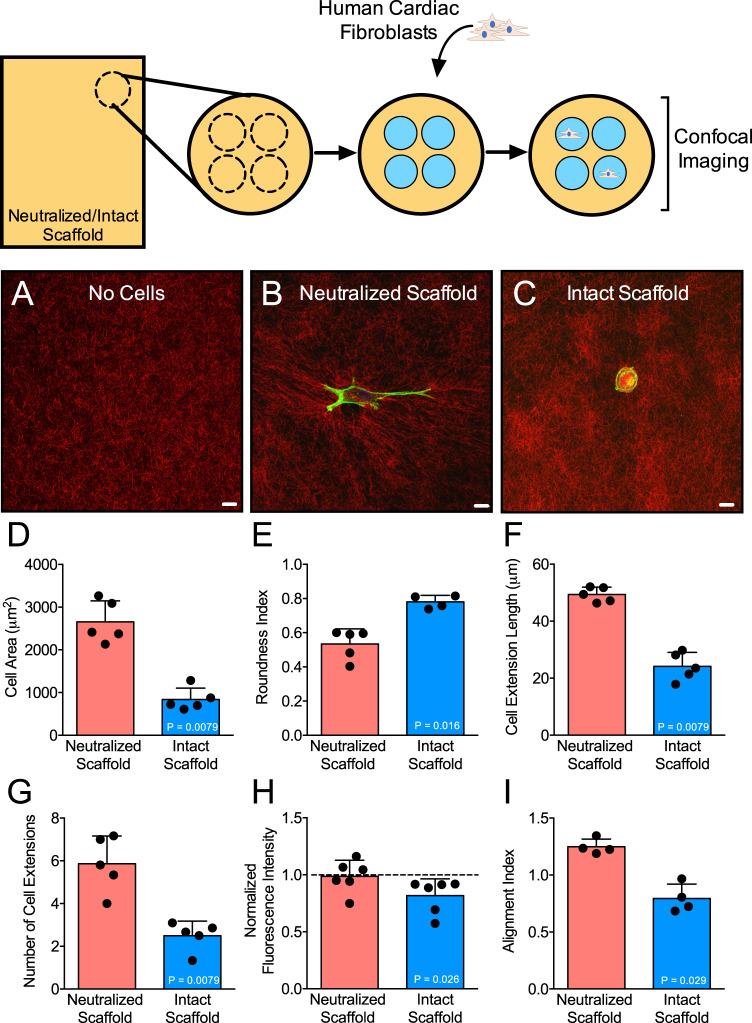
Intact Bioscaffolds Attenuate Human Cardiac Fibroblast Activation and ECM Remodeling. Representative confocal images of matrices embedded within bioscaffolds showing constructs without cardiac fibroblasts (**A**), cardiac fibroblast within intact scaffolds (**B**), and cardiac fibroblast within neutralized scaffolds (**C**). Morphological assessments of cardiac fibroblasts within intact versus neutralized scaffold-matrix constructs are shown (**D–G**; N = 5/group). We assessed alpha-SMA protein expression by flow cytometry and compared cardiac fibroblasts on intact versus neutralized scaffolds. Data is expressed relative to cardiac fibroblasts on tissue culture plastic (H; N = 6/group). Cell-mediated ECM remodeling was quantified by determining the collagen fiber alignment index and compared between matrices embedded with intact and neutralized bioscaffolds (I; N = 4/group). Values are reported as mean ± SD. Statistical significance was determined by the Mann-Whitney test. Scale bar = 20 µm.

We aimed to better understand the bioinductive effects of acellular bioscaffolds on the pro-fibrotic capacity of human cardiac fibroblasts. To allow for assessment of bioinductive effects, we modified a conventional 3D collagen gel model and embedded the cell-matrix construct within acellular biomaterials. We define bioinductive effects as any biological constituents released from the acellular scaffolds into their surrounding microenvironment. With this simple *in vitro* model, we attempted to mimic the conditions *in vivo* wherein surgically implanted scaffolds are not initially in direct contact with the fibroblasts of the infarcted myocardium. Use of a thin gel and single-cell approach allows for direct visualization of the effects of scaffold bioactivity on cardiac fibroblast activation and cell-mediated ECM remodeling (Supplementary Fig. 3). Relative to neutralized scaffolds, human cardiac fibroblasts maintained within intact scaffold-bound collagen microgels show attenuated pro-fibrotic activation as evidenced by a smaller cell size (838 ± 266 µm^2^ versus 2655 ± 492 µm^2^) and more circular cell morphology (0.78 ± 0.04 versus 0.53 ± 0.09) **(**Fig. 2B–E**)**. Cardiac fibroblasts within intact scaffold microgels show fewer cytoplasmic cell extensions (2.5 ± 0.7 versus 5.9 ± 1.3) and shorter cytoplasmic cell extensions (24 ± 5 µm versus 49 ± 3 µm) as compared to cells within neutralized scaffold microgels **(**Fig. 2F,G**)**. We used flow cytometry to measure alpha-SMA protein expression, a phenotypic marker of pro-fibrotic cardiac (myo)fibroblasts. Normalized to cells on plastic tissue culture plates, human cardiac fibroblasts on intact scaffolds show reduced expression of alpha-SMA protein relative to cells on neutralized scaffolds (0.82 ± 0.15 versus 0.99 ± 0.14) (Fig. 2H). Collectively these data indicate that bioscaffolds with preserved bioinductive properties redirect cardiac fibroblasts away from a pro-fibrotic phenotype.

We next assessed the effects of bioscaffolds on cell-mediated remodeling of collagen ECM fibers. Our *in vitro* bioassay allows for cell-mediated ECM remodeling to be directly observed and objectively quantified. This process is recognized as a critical mechanism underlying structural cardiac remodeling and progression to heart failure. Pro-fibrotic fibroblasts when active will remodel, compact, and orient adjacent collagen fibers. At baseline, collagen matrices devoid of cells show a random orientation of collagen fibrils **(**Fig. 2A**)**. Comparatively, matrices with human cardiac fibroblasts embedded within intact scaffolds show less alignment of collagen fibrils as compared to matrices with cells in the neutralized scaffolds (0.79 ± 0.13 versus 1.25 ± 0.06), where we document collagen fibrils to be highly compacted and oriented at the tips of cell extensions **(**Fig. 2I**)**. These results confirm that intact bioactive scaffolds can inhibit pro-fibrotic human cardiac myofibroblast activation and in so doing, attenuate cell-mediated remodeling of structural collagen ECM.

### Bioscaffolds stimulate a pro-vasculogenic paracrine cellular response

**Figure 3:**
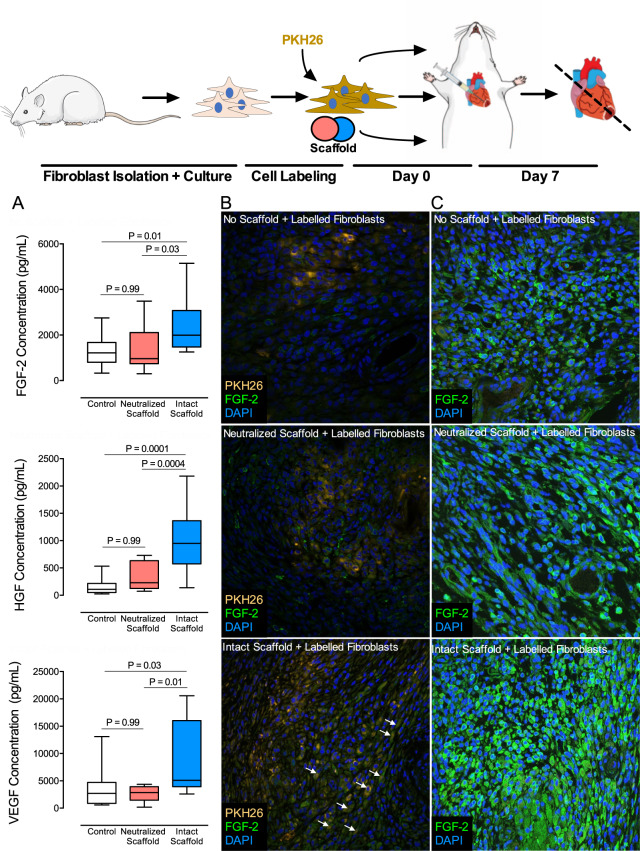
Intact Bioscaffolds Induce a Pro-Vasculogenic Paracrine Response from Human Cardiac Fibroblasts. Protein concentration (**A**) of FGF-2, HGF, and VEGF as assessed by multiplex analysis of the conditioned media is shown from human atrial cardiac fibroblasts on tissue culture plastic (control; N = 10–16), neutralized scaffolds (N = 10–16), and intact scaffolds (N = 10–16). We pre-labelled cardiac fibroblasts isolated from healthy rat hearts with PKH26 and injected labeled cells into injured myocardium immediately after coronary ligation. (**B**) Representative confocal images are shown of injected cells in infarcted myocardium 7 days post-MI for hearts without a scaffold, neutralized scaffold, or intact scaffolds (yellow = PKH26-positive injected cells; green = FGF-2; blue = DAPI). Arrows indicate FGF-2 expression co-localized with injected cells. (**C**) Representative confocal images of infarcted myocardium remote to the fibroblast injection sites. Box plots represent median, and interquartile range. Whiskers represent maximum and minimum values. We calculated statistical significance using one-way analysis of variance (ANOVA). Illustrations were prepared using MindtheGraph software (www.mindthegraph.com) and used under the creative commons license (https://creativecommons.org/licenses/by-sa/4.0/deed.en).

Cardiac fibroblasts can change their secretome in response to signals from the local microenvironment. We next assessed fibroblast paracrine responses at the protein level. We cultured human cardiac fibroblasts on biologic scaffolds and collected conditioned media for multiplex analysis of selected proteins. We have shown that intact scaffolds upregulated key genes associated with blood vessel formation (Fig. 1B). In this experiment we probe conditioned media for the abundance of paracrine factors that are recognized to participate in vasculogenesis and cardiac repair. Relative to neutralized scaffolds, human atrial-derived cardiac fibroblasts on intact scaffolds show substantial increases in expression of pro-vasculogenic FGF-2 (2467 ± 1266 pg/mL versus 1376 ± 957 pg/mL), HGF (1060 ± 623 pg/mL versus 342 ± 256 pg/mL), and VEGF (8266 ± 6974 pg/mL versus 2660 ± 1467 pg/mL) **(**Fig. 3A**)**. We questioned if the observed cardiac fibroblast secretome is specific to the anatomic site of tissue biopsy. We document similar trends in vasculogenic paracrine responses in human cardiac fibroblasts isolated from ventricular myocardium (Supplementary Fig. 4).

### Bioscaffolds promote a reparative vasculogenic cardiac fibroblast phenotype

The paracrine response of cardiac fibroblasts interacting with acellular scaffolds was evaluated *in vivo* using a rat model of human MI by permanent coronary ligation. Animals were randomized to receive an intact scaffold, neutralized scaffold, or no scaffold implant. Prior to scaffold delivery, we injected fluorescently labelled syngeneic rat cardiac fibroblasts into the region of acute infarction immediately following coronary ligation. At 7-days after MI, we observed labeled cardiac fibroblasts to be localized remote from the site of injection towards the intact bioscaffold on the epicardium (Supplementary Fig. 5). Conversely, we observed injected cardiac fibroblasts in animals without scaffolds or neutralized scaffold-treated animals to be contained within the sites of injection. Consistent with the *in vitro* paracrine cell secretome, we noted injected cells to contain a greater abundance of FGF-2 protein when treated with intact bioscaffolds (Fig. 3B**)** as compared to other groups. Additionally, host myocardial cells remote to the site of donor cell injection also show substantial increases in cellular FGF-2 abundance (Fig. 3C). Increased fibroblast FGF-2 was not observed in neutralized scaffold-treated animals or those without scaffolds. We previously established that intact bioscaffolds induce a robust increase of the microvascular network within infarcted myocardium. These observations were documented in a rat ligation model of MI (as used in this study) and a preclinical porcine model of ischemia-reperfusion^14,15^. Using *in vivo* tracking of fibroblasts in this study, we confirm that bioactive properties of intact bioscaffolds support a pro-reparative microenvironment. The redirection of fibroblast phenotypes may help coordinate microvascular assembly.

### Bioscaffolds improve post-MI cardiac functional recovery

**Figure 4:**
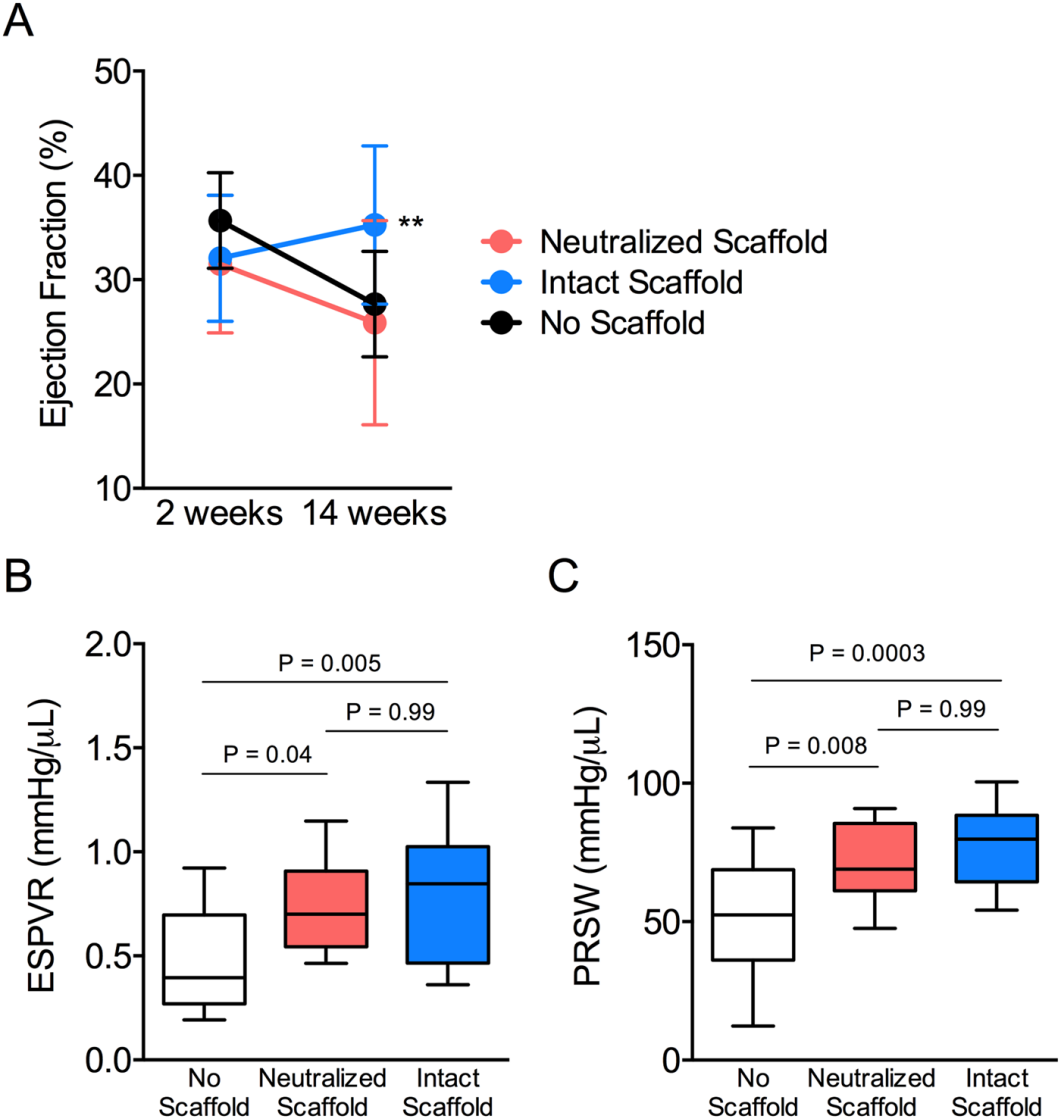
Intact Bioscaffolds Improve Post-MI Functional Recovery. (**A**) Left ventricular ejection fraction is shown from hearts without a bioscaffold (N = 15), neutralized bioscaffolds (N = 16), or intact bioscaffolds (N = 16) as measured by serial echocardiography at 2-weeks (baseline) and 14-weeks after myocardial infarction. We randomized animals to group at 3 weeks after myocardial infarction. We calculated statistical significance using repeated measures two-way ANOVA. We observed group differences for ejection fraction by time (P = 0.007), treatment (P = 0.04), and interaction (P = 0.001). Values are reported as mean ± SD. We performed load-insensitive assessments of cardiac performance using pressure-volume loop analysis. End-systolic pressure volume relationship (ESPVR; **B**) and preload-recruitable stroke work (PRSW; **C**) is reported in animals without scaffolds (N = 15), neutralized scaffolds (N = 17) or intact scaffolds (N = 13). We calculated statistical significance using one-way analysis of variance (ANOVA). Box plots represent median, and interquartile range. Whiskers represent maximum and minimum values.

After showing that bioactive scaffolds can provide a pro-reparative microenvironment to redirect cardiac fibroblast phenotype and activity, we next explored the ability of this adaptive microenvironment to support post-MI functional recovery. We randomized rats with induced MI to receive an intact scaffold, neutralized scaffold, or no scaffold implant. We implanted bioscaffolds 3 weeks following MI to mimic a feasible therapeutic window for human application. We assessed baseline cardiac performance by echocardiography. We obtained baseline measures at 2-weeks after myocardial infarction (1 week before randomization to treatment groups and bioscaffold implantation). Serial measures were repeated at 14-weeks after myocardial infarction. Animals that received intact bioscaffolds show improved left ventricular systolic performance relative to animals without a scaffold or neutralized scaffold-treated animals at 14-weeks (left ventricular ejection fraction: 35.3 ± 7.6% versus 27.7 ± 5.1% and 25.9 ± 9.8%, respectively) **(**Fig. 4A**)**. These data suggest that bioscaffolds can improve functional recovery after MI through a bioinductive mechanism.

Load-insensitive indices of cardiac contractility were assessed by invasive pressure-volume loop analysis using a conductance catheter. Assessments were performed at 14-weeks post-MI and included determination of end-systolic pressure volume relationship (ESPVR) and preload recruitable stroke work (PRSW). Animals treated with intact bioscaffolds show improved ESPVR relative to animals without bioscaffolds (0.80 ± 0.31 mmHg/µL versus 0.49 ± 0.25 mmHg/µL). Hearts with intact bioscaffolds did not show differences in ESPVR as compared to neutralized scaffold-treated hearts (0.80 ± 0.31 mmHg/µL versus 0.74 ± 0.21 mmHg/µL) **(**Fig. 4B**)**. Similarly, animals treated with intact bioscaffolds show improved PRSW relative to animals without bioscaffolds (77.9 ± 14.6 mmHg/µL versus 50.9 ± 21.3 mmHg/µL). Hearts with intact bioscaffolds did not show differences in PRSW as compared to neutralized scaffold-treated hearts (77.9 ± 14.6 mmHg/µL versus 72.2 ± 15.3 mmHg/µL) **(**Fig. 4C**)**. Interestingly, neutralized scaffold-treated animals show substantially improved ESPVR and PRSW relative to animals without scaffold treatments (0.74 ± 0.21 mmHg/µL versus 0.49 ± 0.25 mmHg/µL and 72.2 ± 15.3 mmHg/µL versus 50.9 ± 21.3 mmHg/µL, respectively) indicating that acellular bioscaffolds with or without bioinductive capacity can help maintain systolic cardiac contractility post-MI.

### Bioscaffolds attenuate maladaptive post-MI structural cardiac remodeling

**Figure 5:**
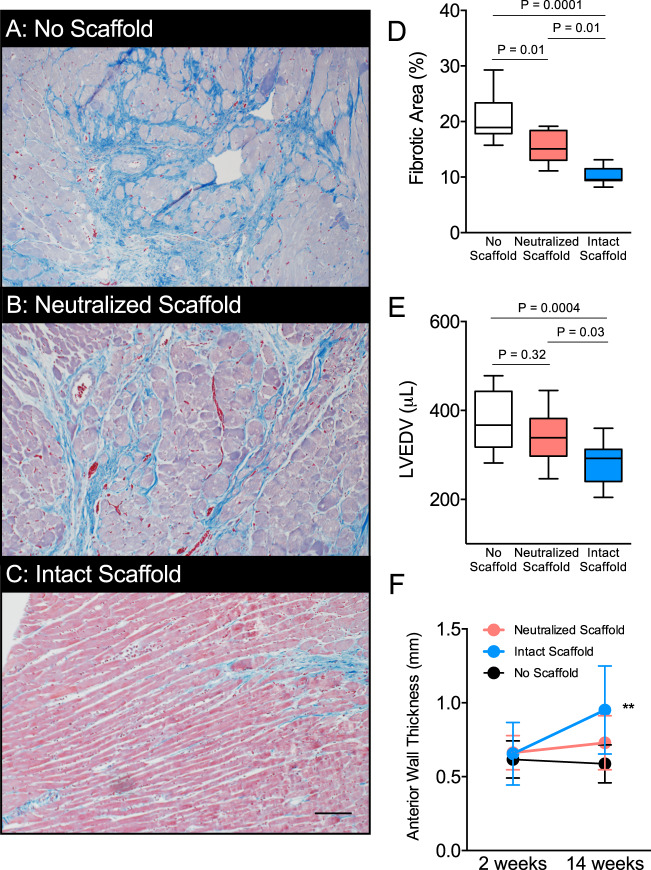
Intact Bioscaffolds Attenuate Post-MI Structural Cardiac Remodeling. Representative images are shown for myocardium stained with Masson’s trichrome from the peri-infarct zone at 14-weeks post-MI for animals without scaffolds (**A**), neutralized scaffolds (**B**), or intact scaffolds (**C**) (blue = collagen, pink = cytoplasm). (**D**) We performed histological analysis to quantify interstitial fibrosis in the peri-infarct zone myocardium for no scaffold (N = 9), neutralized scaffold (N = 9), and intact scaffold (N = 7) groups. Images are shown at 200x magnification (scale bar = 480 μm). We assessed statistical significance using one-way ANOVA. (**E**) We performed pressure-volume loop analysis to assess left ventricular end-diastolic volumes (LVEDV) of no scaffold (N = 12), neutralized scaffolds (N = 15), and intact scaffolds (N = 12). We assessed statistical significance by one-way ANOVA. Box plots represent median, and interquartile range. Whiskers represent maximum and minimum values. (**F**) We assessed cardiac structure using serial echocardiography to measure anterior wall thickness in no scaffold, neutralized scaffold, and intact scaffold treated animals up to 14 weeks after myocardial infarction (N = 15/group). We show a significant difference in anterior wall thickness by time (P = 0.004), treatment (P = 0.0003), and interaction (P = 0.002). We assessed statistical significance using repeated measures two-way ANOVA. Values are reported as mean ± SD.

Maladaptive cardiac tissue remodeling following ischemic injury is a major determinant of progressive heart weakening and decompensation to heart failure^4^. We quantified cardiac interstitial fibrosis using histological analysis with observations focused at the infarct border zone where cell-mediated remodeling and repair is most active. Animals treated with intact scaffolds show reduced myocardial fibrosis 12-weeks after bioscaffold therapy as compared to those without a scaffold or neutralized scaffold-treated animals **(**Fig. 5A–D**)**. We assessed left ventricular end-diastolic volume (LVEDV) using an invasive conductance catheter. LVEDV was reduced for intact scaffold treated animals as compared to those treated with neutralized scaffolds or no scaffolds **(**Fig. 5E**)**. Collectively, these results demonstrate lessening of LV dilation in animals treated with intact scaffolds. Intact bioscaffolds prevented chamber remodeling as LV volume was comparable to reference age and body mass matched healthy hearts^16^.

Loss of viable muscle and progressive scar formation leads to wall thinning. We determined wall thickness as a surrogate measure of infarct scar progression using serial echocardiography. Animals treated with intact scaffolds had thicker anterior walls relative to neutralized scaffold-treated animals and animals without scaffolds **(**Fig. 5F**)**. These data support that intact scaffolds attenuate interstitial fibrosis, LV dilatation, and wall thinning. These parameters of maladaptive structural remodeling pathways are mediated by pro-fibrotic cardiac fibroblasts. Our *in vivo* observations are consistent with human cell model observations that show attenuated fibroblast-mediated ECM remodeling with intact bioscaffolds.

### Human bioscaffold therapy improves post-MI remodeling and functional recovery

Bioscaffolds show therapeutic promise in rodent and porcine preclinical models as observed in this study and others^14,15,17^. In light of these translational data, we performed a first-in-human (Phase 1) observational cohort study to examine outcomes after bioscaffold therapy for human patients with coronary heart disease and acute myocardial infarction. We surgically implanted acellular bioscaffolds as an epicardial patch targeted to the damaged region within 4 weeks of ischemic injury. We used bioscaffold therapy as an adjunct to coronary revascularization (coronary artery bypass grafting) in 8 consenting patients (Supplementary Table 1). Preoperative cardiac MRI determined baseline cardiac structure, function, and myocardial tissue characterization (scar size, presence of microvascular obstruction, and regional tissue perfusion). We defined the location of acute injury by preoperative MRI and provided this information to the surgeon for precise placement of the bioscaffold at the site of injury. We performed serial cardiac MRI (CMR) during study follow-up and compared data from baseline to 6-weeks and 6-months after surgery (Supplementary Table 2).

**Figure 6:**
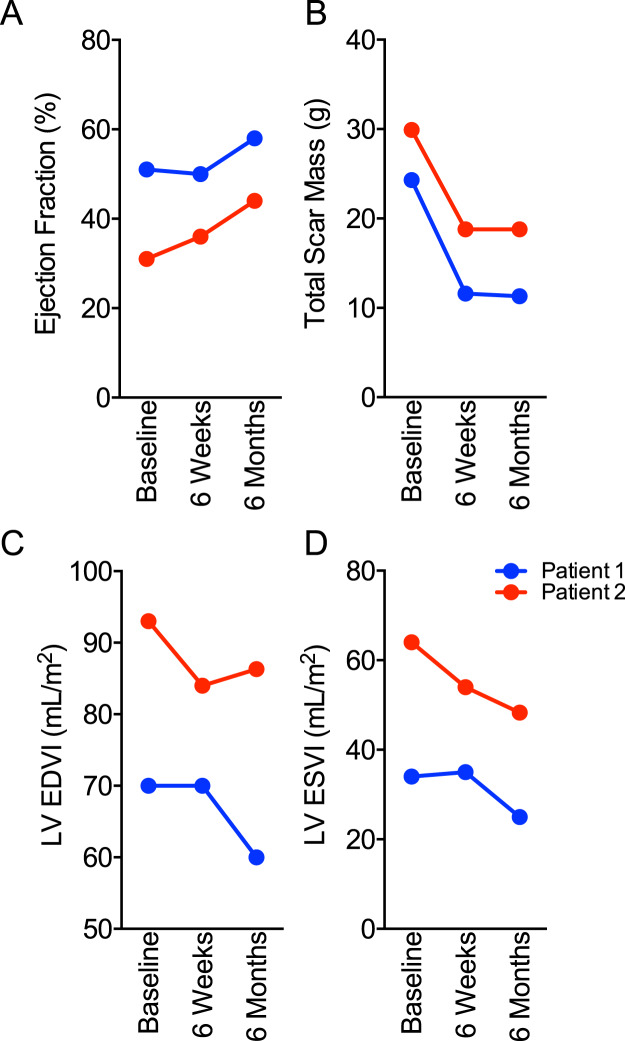
Human Bioscaffold Therapy Improves Post-MI Remodeling and Functional Recovery. We used serial cardiac MRI to assess ejection fraction (**A**), total scar mass (**B**), left ventricular end-diastolic volume index (LVEDVI, **C**), and left ventricular end-systolic volume index (LVESVI, **D**) at baseline (before surgery), 6-weeks post-op, and 6 months post-op for two patients receiving bioscaffold therapy at the time of coronary artery bypass surgery. Both patients (highlighted in blue and red, respectively) had extensive myocardial injuries with microvascular obstruction and reduced pump function at baseline.

Clinical follow-up and imaging was 100% complete. We did not observe any device-related complications or adverse events. We implanted bioscaffolds successfully to the targeted region of ischemic injury in all patients. Using CMR we identified two patients in the cohort with extensive myocardial injury, microvascular obstruction, and depressed global myocardial function. For the first patient, at 6 months following bioscaffold implantation, we show an increase of LV ejection fraction (+8.0%; Fig. 6A) and a decrease of total scar mass (−13.0 g; Fig. 6B) compared to baseline. We document improved structural remodeling at 6 months after injury as a decrease of left ventricular end-diastolic volume index (LVEDVI: −10.0 mL/m^2^, −14.3%; Fig. 6C) and a decrease of left ventricular end-systolic volume index (LVESVI: −9.0 mL/m^2^, −26.5%; Fig. 6D). Similarly, for the second patient we show improved ejection fraction (+13.0%; Fig. 6A**)**, decreased total scar mass (−11.1 g; Fig. 6B), and improved structural remodeling. LVEDVI (−6.7 mL/m^2^, −7.2%; Fig. 6C) and LVESVI (−15.7 mL/m^2^, −24.5%; Fig. 6D) decreased 6 months after injury. We show that human patients with coronary heart disease have clinically important improvements in structural remodeling and functional recovery following bioscaffold therapy.

### Human bioscaffold therapy improves myocardial perfusion of infarcted myocardium

**Figure 7:**
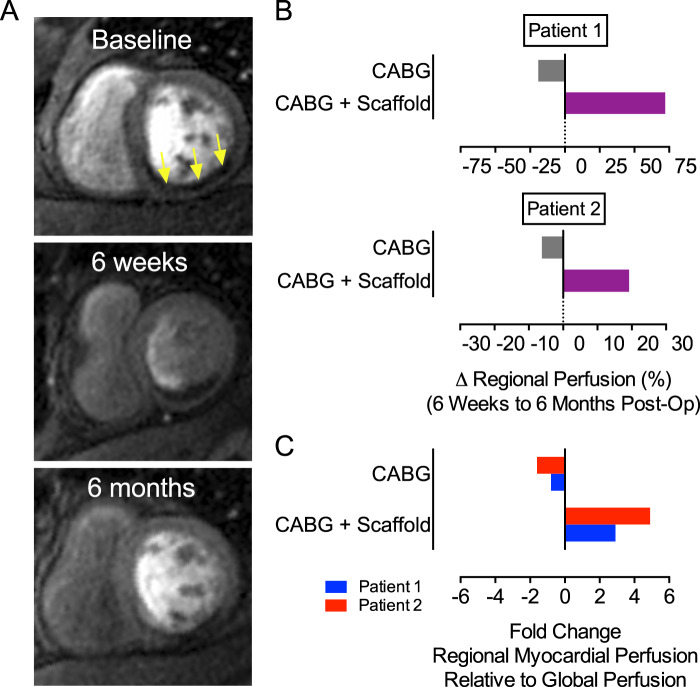
Human Bioscaffold Therapy Improves Myocardial Perfusion of Infarcted Myocardium. (**A**) Representative cardiac MRI images of the left ventricle at baseline, 6 weeks, and 6 months post-op. Arrows indicate site of perfusion defect. (**B**) We illustrate that late tissue perfusion is differentially increased in myocardial regions with implanted bioscaffolds. We quantified regional myocardial perfusion and compared LV regions for each patient between 6 weeks and 6 months post-op. We used the anterior wall as a region with coronary bypass but without bioscaffolds. We used the inferior as a region without coronary bypass but with bioscaffold implantation. (**C**) We compared regional myocardial perfusion relative to global LV perfusion for both patients between 6 weeks and 6 months post-op.

Our preclinical studies documented a marked enhancement of microvascular networks within infarcted myocardium following treatment with bioscaffolds^14,15^. To explore this effect in human patients we assessed tissue perfusion by CMR and performed a relative comparison at each time point (Fig. 7A). We anticipated that improvements in perfusion attributed to surgical coronary revascularization could be documented and quantified by early changes in perfusion - between baseline and 6 weeks post-op (early follow-up). We predicted that perfusion improvements attributed to neovascularization and a restored microvasculature could be shown by later changes in perfusion - between 6 weeks and 6 months post-op (late follow-up). This analysis assumes that coronary inflow attributed to surgical revascularization will not substantially increase late after revascularization. Myocardial perfusion assessments by region and time are summarized in Supplementary Table 3.

We compared the magnitude of regional tissue perfusion between two anatomic regions using each patient as their own control. We used the mid-anterior wall to represent a region with surgical revascularization but without bioscaffolds. We used the inferior wall to represent a region of combined surgical revascularization and bioscaffold therapy. At the late assessment period, we show enhanced perfusion in the region underlying the bioscaffold (+72.1%) as compared to the area that received CABG alone (−19.1%) for patient 1. Similarly, we show increased perfusion at the site of bioscaffold implantation (+19.2%) as compared to the area that received CABG alone (−6.2%) **(**Fig. 7B) for patient 2. We illustrate that late tissue perfusion is differentially increased in myocardial regions with implanted bioscaffolds. We note this observation at late follow-up when the perfusion effects of coronary bypass are mature and not expected to increase.

We next examined regional myocardial perfusion in the context of global perfusion for the whole heart. Global perfusion reflects the entire myocardial tissue mass and was observed to be consistently increased for both patients at each time point. We show increased global perfusion 6 weeks post-op as compared to pre-op baseline measures (+0.46 mL/min/g) for patient 1. Further improvement was observed between 6 weeks and 6 months post-op (+0.25 mL/min/g). Similarly, global perfusion for patient 2 increased between 6 weeks post-op and baseline (+0.50 mL/min/g) and slightly increased between 6 months post-op and 6 weeks post-op (+0.05 mL/min/g; Supplementary Table 3). We used these global perfusion metrics to control for possible confounding effects of coronary bypass on regional perfusion changes. We compared myocardial perfusion in the anterior (CABG without bioscaffold) and inferior (CABG with bioscaffold) myocardial regions at late follow-up and expressed regional perfusion relative to concomitant changes in global LV perfusion. Patient 1 and patient 2 show a 2.9 and 4.9-fold relative increase respectively, for myocardial perfusion in regions underlying the bioscaffold implants (Fig. 7C). Conversely, we show a 0.8 and 1.6-fold respective relative decrease in myocardial perfusion for regions that received CABG without a bioscaffold. This analysis further supports that bioscaffolds can enhance tissue perfusion in human patients after myocardial infarction.

Although this clinical observational study was not designed to validate therapeutic effectiveness, the results of our first-in-human study offer the possibility that acellular bioscaffold therapy may improve regional perfusion, decrease infarct scar, and reverse structural remodeling after ischemic injury. Our comparative analysis by region suggests that bioscaffolds may provide benefits over and above those afforded by surgical coronary revascularization alone. Importantly, these clinical data are consistent with our observations recorded using *in vitro* human cell models and *in vivo* animal models of human disease.

## Discussion

Using acellular biomaterials with bioinductive properties, we propose a clinically feasible strategy to optimize cardiac repair during surgical revascularization procedures. Bioactive scaffolds can stimulate pathways not directly targeted by any standard of care therapies. Bioscaffold therapy may be capable of addressing an unmet clinical need for improved functional tissue recovery after ischemic myocardial injury. Here, we show that acellular bioscaffolds can redirect cardiac fibroblast functions within infarcted heart muscle. Pro-fibrotic activity was attenuated while a vasculogenic secretome was stimulated. Critical paracrine mediators of blood vessel assembly were released. We previously documented that paracrine mediators released from such cell-bioscaffold interactions can support new blood vessel formation and stimulate adaptive cardiac repair^14,15^. Using *in vitro* cell-based assays and preclinical animal models, we illustrate that bioinductive properties of the biomaterial are critical for fibroblast redirection. We observed improved tissue structure and function in a rodent model of myocardial infarction and a small series of human patients with acute ischemic injury.

We and others define clinically important reverse remodeling as a greater than 10% reduction in LV volume at 6 months after infarction. Reverse remodeling is not anticipated for patients with extensive infarction and microvascular obstruction^18^. In our observational study, the two subjects with the most extensive tissue injury and microvascular obstruction showed a greater than 24% reduction in LVESVI in association with bioscaffold therapy at 6 months after infarction. Reduced LVESVI is strongly predictive of an improved long-term prognosis after myocardial infarction. Improved LVESVI after infarction can be used to identify potential therapies that can reduce mortality, sudden cardiac death, and heart failure^19,20^. Reverse remodeling is likely a consequence of attenuated fibroblast-mediated maladaptive tissue remodelling.

In addition to reverse structural remodeling, we show improved late perfusion in myocardial regions targeted with bioscaffold therapy. Bioscaffolds may uniquely stimulate repair of the downstream microvascular network after damage by injury. Coronary grafting restores coronary artery inflow but cannot perfuse damaged tissues with a deficient microvasculature. Microvascular disease is common and can result in incomplete revascularization after coronary bypass surgery with poor functional recovery. Coronary grafting and bioscaffolds in combination may be synergistic and by facilitating optimal tissue perfusion deep within injured tissues functional recovery may be improved.

Cardiac fibroblasts represent a small proportion of total cardiac cells but the number of fibroblasts increases ten-fold after an ischemic injury^3,21^. The existing paradigm dictates that cardiac fibroblast proliferation and expansion replaces lost myocardium with collagenous scar. Pro-fibrotic fibroblast activation can persist long after complete resolution of the initial ischemic injury^22^. Persistent fibroblast activity causes tissue fibrosis and is considered maladaptive once infarcted tissues are healed as a mature scar. Supporting that fibroblasts can induce functional mechanisms of repair, Ubil and colleagues show that a subset of cardiac fibroblasts can participate in neovascularization early after ischemic injury^7^. Supporting that fibroblast activities can be redirected, Pillai and co-workers provide data to indicate that local microenvironments can instruct the fate that fibroblasts adopt after injury^8^. Plikus and associates identified a microenvironment that can reprogram pro-fibrotic dermal fibroblasts towards a non-fibrotic fate and create “scarless” wound healing^9^. The results of our study further support the concept that fibroblasts are plastic and can be therapeutically redirected to promote an adaptive and non-fibrotic repair that can recover tissue function lost to injury.

Badylak proposed a pragmatic concept that acellular ECM biologic scaffolds can be used to modify endogenous cell behaviors at sites of injury^23^. Building on his work and others, we show that acellular bioscaffolds can redirect cardiac fibroblasts towards a pro-vasculogenic state by way of bioinductive properties. To date, directed and intentional control of cardiac fibroblast phenotypic plasticity has focused on using techniques of genetic reprogramming. Although this approach offers the promise of regenerating lost myocardium with new muscle cells, adequate reprogramming efficiency *in vivo* has yet to be achieved^24^. Nevertheless, cardiac fibroblasts remain an attractive target for tissue engineering given their proliferation after injury and inherent propensity to participate in repair^5^. We believe that acellular bioscaffolds can be used to target cardiac fibroblasts and change their function without the translational hurdles that challenge genetic manipulation or other cell-based modalities.

The bioinductive mechanisms by which acellular ECM bioscaffolds confer their effects on heart cells are not fully understood. Given its composition, the ECM serves as a reservoir for numerous bioactive factors that can direct cell behaviour^25^. FGF-2 has a known cardioprotective role in the heart, with potent vasculogenic and anti-fibrotic properties^26–28^. In prior studies, we identified that FGF-2 bound within acellular bioscaffolds is necessary to induce pro-vasculogenic signalling in human cardiac fibroblasts. Blocking FGF signalling receptors in cardiac fibroblasts or limiting bioavailability of FGF-2 via chemical cross-linking of the bioscaffolds blocked the pro-vasculogenic response and associated functional benefits to the post-MI heart^14^. Similar results were observed in the present study after chemically extracting FGF-2 and other factors from the bioscaffolds. Interestingly, bioinductive properties were found to be beneficial in stimulating functional recovery but were not essential. Other properties of the ECM scaffold were also important to functional tissue repair. These intriguing data highlight that endogenous cardiac repair is a symphony performed by an ensemble and not a solo act. It is unlikely that this complex multifaceted process can be recapitulated using isolated single gene, protein, or cell therapies. A platform that leverages a biologic matrix microenvironment is critical to induce functional repair. In addition to growth factor signalling, the release of matrix-bound nanovesicles and chemotactic ECM degradation products are other possible mechanisms that may mediate the benefits of acellular ECM scaffold cardiac repair^12,29^. Biomechanical restraint on the epicardium with use of scaffolds may also stimulate pathways of repair.

Neovascularization of ischemic tissue has been associated with improved functional recovery over time^30^. Intramyocardial delivery of exogenous pro-vasculogenic growth factors, such as FGF-2 or VEGF, did not show substantial clinical benefits after translation from animal models to human patients^31,32^. Providing a sustained paracrine response may be critical as protein, gene, or cell therapies have proven to provide only transient effects and have largely failed in human translation. Gene, protein, or cell-based therapeutic strategies may not be able to maintain local concentrations of key vasculogenic agents over a prolonged period of time. Instructive scaffolds that directly target endogenous cell paracrine responses may achieve a similar and more sustained boost in myocardial growth factors^33^. Importantly, we previously documented increased myocardial FGF-2 protein abundance as late as 14-weeks following surgical implantation of acellular bioscaffolds^14^. Accordingly, our translational work highlights the feasibility of using acellular bioscaffolds to induce sustained responses that support effective neovascularization of ischemic tissues.

Infarct scar is composed of several different cell types. It is likely that acellular bioscaffolds interact with and modulate additional resident cells beyond fibroblasts. Recruitment of progenitor cells and polarization of activated macrophages towards a pro-reparative phenotype has been demonstrated in other forms of acellular bioscaffold mediated repairs^29^. The ability of acellular bioscaffolds to recruit and modify inflammatory and progenitor cells in conjunction with cardiac fibroblasts in cardiac scars will be the subject of future study.

We identify study limitations that should be noted. First, functional cardiac recovery after injury is mediated by multiple endogenous pathways of repair. Using preclinical models, it is difficult to isolate the individual contributions of specific pathways on observed functional gains. For example, we cannot quantify how much recovery of cardiac structure and function is a direct result of observed decreases in fibrosis versus concomitant increases in vasculogenesis nor can we determine interactive effects between these variables. Further, it is difficult to ascertain scaffold effects on native cardiac fibroblasts in our model as PKH26 cell-labelling has been known to leach into surrounding cell types within the tissue^34^. Second, our first-in-human observational clinical study was not adequately powered to validate therapeutic effectiveness. Our clinical observations were also limited by only two patients showing extensive tissue injuries before therapy. While efforts were made to compare regions, the contribution of surgical revascularization on functional recovery cannot be completely isolated without further clinical investigations using a randomized study design.

Surgical revascularization is performed worldwide in over 800,000 patients per year^35^. Many patients have microvascular disease leading to incomplete revascularization after coronary bypass procedures. Bioscaffold therapy as an adjunct to coronary bypass surgery may offer a new frontier to optimize surgical revascularization for patients with coronary heart disease. More broadly, leveraging the instructive nature of ECM bioscaffolds to redirect fibroblasts is an exciting prospect that may provide benefits to numerous tissues and organ systems at risk of maladaptive fibrosis, microvascular damage, and loss of biomechanical function.

## Materials and Methods

### Experimental study design

#### Human fibroblast cell models

We used *in vitro* cell modeling to determine the role of bioactive constituents within acellular bioscaffolds on human cardiac fibroblast gene and protein expression, pro-fibrotic activation, and capacity to remodel collagen ECM components. Human cardiac fibroblasts were isolated from cardiac muscle (right atrial appendage). To enhance translational relevance, we obtained human fibroblasts from patients with coronary heart disease undergoing surgical revascularization procedures. We obtained human ethics approval from the Conjoint Health Research Ethics Board at the University of Calgary. Patients provided informed consent. Cardiac fibroblast function and phenotype was assessed using RNA sequencing, flow cytometry, and multiplex protein analysis. The fibrotic capacity of human cardiac fibroblasts was investigated using a novel bioassay that employs a thin collagen gel embedded within acellular scaffolds.

#### Preclinical model of human disease

We used a preclinical rodent model of myocardial infarction that is well-established and clinically relevant to determine the role of bioscaffolds and the specific contribution of retained bioactive properties on parameters of cardiac repair^36^. Myocardial infarction was induced in rats by permanent coronary ligation. Experimental animals were randomized to group. Acellular bioscaffolds were implanted 3 weeks after MI (to mimic a feasible clinical therapeutic window) and followed for 14-weeks to assess post-MI remodeling and functional recovery. Investigators were blinded to group for assessment of study end-points. Functional and structural analysis was performed using echocardiography and conductance catheter derived pressure-volume loop analysis. Histology and immunohistochemistry of cardiac tissues was performed. We received appropriate approvals from our institutional animal care committee to perform these experiments.

#### Clinical observational study

We performed a non-randomized, unblinded and open-label Phase 1 clinical observational study to determine clinical feasibility, safety and physiologic effects of bioscaffold therapy in human patients. Bioscaffolds were implanted in 8 patients within 4 weeks of acute myocardial ischemic injury requiring surgical revascularisation. Clinical effects of acellular bioscaffolds was determined by serial CMR imaging with analysis up to 6 months after the procedure. We obtained human ethics approval from the Conjoint Health Research Ethics Board at the University of Calgary. The study was conducted in compliance with the Declaration of Helsinki. Patients provided informed consent. We obtained a letter of no objection from Health Canada for investigational use of bioscaffolds. Inclusion and exclusion criteria are reported online (ClinicalTrials.gov Identifier: NCT02887768; Registration Date: 02/09/2016). No statistical method was employed to determine a preliminary sample size as this study was first-in-human.

### Preparation of Bioscaffolds

We used commercially available (CorMatrix-ECM; CorMatrix Cardiovascular Inc., GA, USA) porcine small intestinal submucosal extracellular matrix (SIS-ECM) biomaterial to create acellular bioscaffolds. This FDA-approved device is processed to remove resident cells (decellularized) while retaining its native 3-D collagen architecture and embedded bioactive proteins. SIS-ECM has been shown to retain FGF-2, VEGF, glycosaminoglycans, fibronectin, laminin, and other key ECM-based matricellular signaling biopeptides. 7 ×10-cm decellularized ECM sheets were cut into 1-cm diameter circles using a tissue biopsy punch (Acu-Punch, Acuderm Inc., FL, USA). The intact SIS-ECM scaffold was rehydrated with phosphate buffered saline (PBS; Lonza, MD, USA). Neutralization of the bioactive constituents in the ECM scaffold was achieved by dissociating and denaturing said ECM-adsorbed constituents with an overnight soak at room temperature in 4 M guanidine hydrochloride. Treated scaffolds were washed three times at 5 minutes per wash to ensure removal of residual guanidine hydrochloride prior to cell experimentation.

### Biochemical characterization of bioscaffolds

FGF-2 has been previously identified by our group as a major bioactive constituent in intact SIS-ECM scaffold preparations and was therefore chosen as a surrogate marker to evaluate the efficacy of the neutralization protocol^14^. Intact and neutralized ECM scaffolds were incubated in serum-free media (SFM; IMDM; Lonza, MD, USA) overnight at 37 °C in 5% CO_2_ in the absence of cells. The resultant conditioned media was analyzed by multiplex assays (Eve Technologies, Calgary, Canada) to quantify the concentration of FGF-2 passively released from the ECM scaffolds.

### Biomechanical characterization of bioscaffolds

1.2 ×1.2 cm square patches were cut from either intact or neutralized ECM scaffolds. Samples were pre-hydrated for 10 minutes prior to testing using PBS. Samples were mounted onto a biaxial testing machine (ElectroForce Systems, TA Instruments, MO, USA) that stretches the tissue simultaneously in two orthogonal directions by attaching two suture lines to each side of the patch using four fish hooks per side. Dots were drawn on the surface of the patch material in a prescribed pattern and tracked with the camera for local strain measurements. Samples were immersed in PBS at 37 °C and pre-loaded to the range of 0.05–0.07 N. Samples were then tested using displacement-controlled loading to 40% strain for 10 loading/unloading cycles. The first 9 cycles served as preconditioning and the last cycle was used to generate the strain-stress curves. Test protocols were carried out in sequence at peak displacement ratios of 1:1, 1:0.75, 1:0.5, 0.5:1, and 0.75:1 for each sample. At the end of the experiment, the 1:1 protocol was repeated to detect if there was any damage to the sample.

### Human Cardiac fibroblast isolation and expansion

Human cardiac fibroblasts were isolated from right atrial appendage (N = 16) or left ventricular core biopsies (N = 3) taken from consenting patients undergoing cardiac surgery at Foothills Medical Center (Calgary, Alberta) as previously described^14^. All experiments involving human tissue were approved by the Conjoint Health Research Ethics board at the University of Calgary and conform to the Declaration of Helsinki. Samples were processed into 0.5–1 mm pieces and suspended in Iscove’s Modified Dulbecco’s Medium (IMDM; Lonza, MD, USA) supplemented with 10% fetal bovine serum (Gibco by Life Technologies, ON, Canada) and 50,000 units of penicillin-streptomycin (Life Technologies, ON, Canada). Tissue suspensions were plated on tissue culture dishes coated with 0.1% gelatin and cultured at 37 °C in 5% CO_2_. Passages 1–4 were used for experiments. All cells were serum starved for 24-hours prior to experimental use.

### Assessment of cell viability by flow cytometry

Cell apoptosis and necrosis was assessed using a FITC Annexin V apoptosis detection kit (BD Biosciences, ON, Canada). Human cardiac fibroblasts were harvested from experimental constructs as described above. Cell pellets were resuspended in 1x Annexin V binding buffer with propidium iodide (1:100 dilution) and FITC Annexin V (1:100 dilution) before flow cytometry analysis.

### RNA Sequencing analysis

Total RNA was isolated from human cardiac fibroblasts seeded on tissue culture plastic, intact ECM scaffolds, and neutralized ECM scaffolds using RNeasy Mini Kit (Qiagen, Germany), per manufacturer’s instructions. RNA quality was assessed with the Agilent 2200 Tapestation RNA (RIN) assay (Agilent, CA, USA). 30 µL of total RNA per sample were used for cDNA library preparation [TruSeq Stranded mRNA Library Preparation (Illumina, CA, USA). RNA sequencing data (535 M reads) was generated using a 75 cycle high-output kit on an Illumina NextSeq500 (Illumina, CA, USA). RNA sequencing reads pseudoaligned to the human NCBI RefSeq transcript database dated January 2017, using Kallisto 0.42.4^37,38^. Sleuth was used for differential gene expression using a linear model containing two terms: the nominal scaffold factor and the patient from which each sample was derived^39^. Genes passing the Likelihood Ratio Test with Benjamin-Hochberg (false discovery rate) corrected p-values and Wald test (<0.05) were considered differentially expressed. Differentially expressed genes were annotated and analyzed for enrichment using Ingenuity Pathway Analysis (Qiagen, CA, USA).

### Preparation of bioscaffold-microgel model to assess cardiac fibroblast activation

A novel floating 3-D collagen model was used to assess individual cardiac fibroblast activation and ECM remodeling capacity when seeded on acellular scaffolds (Supplementary Fig. 3). The model was developed from a foundation of prior work^40,41^. Using a tissue biopsy punch (Acu-Punch, Acuderm Inc., FL, USA), six 1-mm diameter holes were made in either the intact or neutralized scaffold constructs. Type I bovine dermal collagen (Advanced Biomatrix, CA, USA) was diluted to a working concentration of 1.0 mg/mL and pH ~ 7.4. The collagen solution was poured onto a hydrophobic surface created by parafilm-coating a tissue culture dish. The hole-punched scaffold constructs were placed carefully on top of the collagen, allowing the solution to fill the openings. ECM constructs were incubated for 2 hours at 37 °C in 5% CO_2_ to ensure proper collagen polymerization. The resultant collagen-acellular scaffold microgels were detached from the tissue culture dish using warm PBS, inverted, and floated in serum free IMDM. Human cardiac fibroblasts were then seeded at low density (~2,500 cells per construct) and maintained overnight. Low cell density was used to ensure imaging of single cells and their effects on the local ECM without the confounding remodeling effects of neighboring cells.

### Assessment of cell morphology in the bioscaffold-microgel model

Cell-bioscaffold microgel constructs were imaged as previously described^26,40^. In brief, constructs were fixed in 4% PFA and permeabilized using 0.1% Triton-X. Cell actin cytoskeleton was stained using Alexa Fluor 488 phalloidin (Life Technologies, ON, Canada) and nuclei were marked with DAPI. Constructs were imaged using a confocal laser microscope (LSM 5, Carl Zeiss, Germany). Morphological assessments included: cell extension length, number of cell extensions, cell area, and cell roundness (where a value of 1.0 indicates a perfect circle). Cell extension length was assessed from cell center to the tip of the extension using ImageJ image analysis software (Ver. 1.50, NIH, USA). Multi-Cell Outliner (http://rsbweb.nih.gov/ij/plugins/multi-cell-outliner.html) ImageJ plug-in was used to quantify cell roundness and cell area.

### Quantification of ECM Remodeling in the bioscaffold-microgel model

ECM remodeling was defined using a collagen fiber alignment index as previously described^40,41^. Collagen was visualized using confocal reflectance microscopy and the fibers at the tips of the cell extensions were used to assess ECM remodeling. After selecting the area at the tip of the cell extensions, the Fast Fourier Transform (FFT) function was used to convert the original image to its frequency domain. To quantify the distribution of collagen fibers at a given angle, pixel intensities were summed along a straight line from the image center at intervals between 0° and 180° using Oval Profile ImageJ plug-in (http://rsb.info.nih.gov/ij/plugins/oval-profile.html) producing a pixel intensity value for each angle. From the resultant pixel intensity plot, the collagen fiber alignment index was defined by calculating the area under the curve within ±10° of the maximum intensity value.

### Assessment of alpha-SMA expression by flow cytometry

Bioscaffold sheets were cut into 3.5 cm diameter circles to cover the bottom of a well in a standard six-well tissue culture plate. Human cardiac fibroblasts were seeded at a density of 600,000 cells/group in serum-free IMDM and constructs were incubated at 37 °C, 5% CO2. After 72 hours, cell pellets were harvested from each construct and resuspended in polystyrene round bottom tubes using 0.25% trypsin-EDTA. Cells were fixed in ice-cold methanol at −20 °C, followed by washing with PBS and 1% bovine serum albumin (BSA). Cells were permeabilized with 1% Triton-X and collected by centrifugation. Cells were stained with anti-alpha-SMA conjugated with FITC (1:300 dilution; Sigma-Aldrich) and resuspended in PBS for flow cytometry analysis.

### Paracrine response of human cardiac fibroblasts to bioscaffolds

One hundred thousand isolated cardiac fibroblasts were seeded onto circular (10-mm diameter) bioscaffolds (or cell culture plastic as a control) and incubated in serum-free IMDM at 37 °C in 5% CO_2_. After 24 hours, conditioned media from the experimental groups was collected, labelled alpha-numerically, and sent for blinded analysis to quantify the concentration of FGF-2, HGF and VEGF by multiplex analysis (Eve Technologies, AB, Canada).

### Experimental animals

The experimental protocol was approved by the University of Calgary Animal Care Committee (ACC). All procedures performed were in accordance with the Canadian Council on Animal Care. 150–200 g Male rats (Fischer CDF® strain) were obtained from Charles River Canada Inc. (QC, Canada).

### Rat myocardial infarction model

Animal handling and myocardial infarction was performed as previously described^14^. In brief, animals were anesthetized and maintained with isoflurane for the duration of the surgical procedure. Using left anterolateral thoracotomy to expose the heart, the left anterior descending artery was identified and subsequently ligated. The incision was re-approximated and animals were recovered.

### Surgical implantation of bioscaffold

Infarcted animals were randomly assigned to one of three treatment groups: 1) no scaffold, 2) intact scaffold implant, or 3) neutralized scaffold implant (N = 16 per treatment group). The treatment procedure was performed 3-weeks post-MI. The heart was exposed by left anterolateral thoracotomy one intercostal space caudal to the previous incision and the infarcted region was identified visually. Intact or neutralized scaffolds were sewn onto the epicardial surface of the infarct using a continuous 7–0 polypropylene suture. Animals in the no scaffold group underwent a similar suture technique however without the placement of an acellular scaffold.

### Labeled cardiac fibroblast injections in rodent model of myocardial infarction

Cardiac fibroblasts were isolated from healthy male rat hearts similarly as described above. Prior to surgery, isolated cardiac fibroblasts were stained with a PKH26 red fluorescent cell linker kit for general cell membrane labeling (Sigma-Aldrich, ON, Canada) as per the manufacturer’s instructions. Male rats (Fischer CDF® strain) were anesthetized and MI was induced by LAD ligation via left anterolateral thoracotomy as previously described. Immediately after MI, a total of 2.0 ×10^6^ labelled cardiac fibroblasts in 200 µL of PBS were injected into the infarcted myocardium at four different sites (50 µL/injection) using a 29-gauge needle. Animals were maintained for 7-days after which hearts were perfusion-fixed with 10% neutral buffered formalin and explanted. Specimens were then washed with PBS and dehydrated in 10%, 20%, and 30% sucrose solution before OCT embedding and cryosectioning. 10 µm sections were cut and stored in a −80 freezer.

### Animal sacrifice and explant

Animals were sacrificed 14-weeks post-treatment by lethal injection of 20 mM potassium chloride under anesthesia. The LV of explanted hearts were isolated and divided in long axis through the infarcted anterior myocardial wall. LV specimens were fixed in 10% NBF (Neutral Buffered Formalin; VWR International, PA, USA) and embedded in paraffin for histological analysis.

### Assessment of cardiac fibrosis

Paraffin-embedded LV specimens were stained with Masson’s Trichrome to visualize collagen fibrils (blue). Fibrosis was quantified using ImageJ as the relative area of blue staining averaged across 5 images per LV sample in the peri-infarct area. In the treatment group of animals, implanted scaffolds were not included in the area of assessment.

### Labelled cardiac fibroblast cell tracking & immunofluorescence staining

Cryostat sections were thawed at room temperature and air dried overnight. Slides were then rehydrated in PBS for 15 minutes. To optimize PKH26 fluorescent signal and viewing of PKH labelled cells, sections were directly stained with 4’6-diamino2-phenylindol (DAPI) after rehydration and mounted with an anti-fade mounting media.

For anti-FGF-2 immunofluorescence staining, antigen retrieval was performed by incubating slides in 10 mM citric acid in 0.05% tween-20 at a pH of 6.0 for 20 minutes at 95–100 °C, followed by a cool-down at room temperature for 20 minutes. Sections were permeabilized and blocked with 1% goat serum 0.1% Triton X-100 in PBS for 20 minutes, stained using primary anti-FGF-2 antibodies (rabbit polyclonal; Santa Cruz Biotechnology, CA, USA). Stained specimens were examined using a confocal laser microscope (LSM 5, Carl Zeiss, Germany).

### Echocardiography

Echocardiographic evaluation was performed as previously described^14^. In brief, animals received echocardiography 2-weeks post-MI (baseline prior to treatment), and 14-weeks post-MI. All echocardiograms were performed under isoflurane inhalational anesthesia in the dorsal decubitus position, and recorded using an Esaote MyLab30 Gold Cardiovascular Ultrasound system (Canadian Veterinary Imaging, ON, Canada). Animals with an ejection fraction of >50% were not randomized into treatment groups and excluded from experimentation.

### Invasive cardiac pressure-volume loop analysis

Cardiac structure and load independent indices of cardiac function were assessed using a pressure volume loop system at the terminal experimental endpoint (14-weeks post-MI). A 2 F conductance catheter (SPR-838 or SPR849, Millar Instruments, TX, USA) was inserted into the LV chamber via the right carotid artery. Left ventricular pressure and volume data was collected and analyzed as previously described using ADInstruments software (CO, USA)*(16)*.

### Patient selection and enrolment

This study protocol was approved by both the Conjoint Health Research Ethics board at the University of Calgary and Health Canada. All eight patients provided written, informed consent. Patients eligible for the study were those diagnosed with MI (STEMI or NSTEMI) and were within three weeks of receiving CABG surgery for the acute event. Patients with a confirmed diagnosis of MI and indications for CABG were further screened using CMR to confirm the presence of non-viable myocardium in the anterior wall of the LV and exclude the presence of non-viable myocardium or scar within other myocardial territories (ClinicalTrials.gov Identifier: NCT02887768).

### CMR Image acquisition and analysis

Serial cardiac magnetic resonance (CMR) imaging was performed at baseline (pre-op) as well as 6-weeks and 6-months post-treatment using a 3.0-tesla MRI scanner (Avanto; Siemens Healthcare; Erlangen, Germany). Imaging was performed at the Stephenson Cardiac MR Center (Calgary, Alberta). Images were acquired using CINE, late gadolinium enhancement (LGE) and T1-mapping by saturation recovery single-shot acquisition (SASHA) protocols^42^. CMR images were analyzed using cvi42 Software (Circle Cardiovascular, AB, Canada). Resting myocardial perfusion was calculated from CMR images using Medis Research software (Leiden, The Netherlands).

### Statistical analysis

All data are expressed as mean ± SD of at least three independent experiments. Prism 5.0d (GraphPad Software Inc., CA, USA) statistical software was used for all statistical analysis except raw RNA sequencing data (further described in supplementary materials). Data was analyzed by unpaired Student’s test when comparing two samples; analysis of variance (ANOVA) when comparing multiple samples. Post-hoc comparisons were analyzed with Bonferroni’s test. Experiments with a small sample size were analyzed using non-parametric statistical tests. P values <0.05 were considered significant. Two-tailed tests were used unless otherwise specified.

## Supplementary information

Supplementary Data.

## Data Availability

The data that support the findings of this study are available from the authors on reasonable request, see author contributions for specific data sets.
