# Supplementary material for: Acellular bioscaffolds redirect cardiac fibroblasts and promote functional tissue repair in rodents and humans with myocardial injury
Source: Sci Rep. 2020 Jun 11;10:9459. doi: 10.1038/s41598-020-66327-9 (PMC7289874; doi:10.1038/s41598-020-66327-9)
Supplement: Supplementary file 1 — Supplementary Data. [file 41598_2020_66327_MOESM1_ESM.docx]

**Acellular bioscaffolds redirect cardiac fibroblasts and promote functional tissue repair in rodents and humans with myocardial injury**

Daniyil A. Svystonyuk, BSc^1^; Holly E.M. Mewhort, MD PhD^1^; Ali Fatehi Hassanabad, MD MSc^1^; Bobak Heydari, MD MPH^2^; Yoko Mikami, MD PhD^2^; Jeannine D. Turnbull, BSc^1^; Guoqi Teng, PhD^1^; Darrell D. Belke^1^, PhD; Karl T. Wagner, BSc^1^; Samar A. Tarraf, BSc^3^; Elena S. DiMartino, PhD^3^; James A. White, MD^2^; Jacqueline A. Flewitt, MSc^2^; Matthew Cheung, MD^2^; David G. Guzzardi, BSc^1^; Sean Kang, BSc^1^; Paul W.M. Fedak^1^*, MD PhD

^1^Section of Cardiac Surgery, Department of Cardiac Sciences, Cumming School of Medicine, Libin Cardiovascular Institute of Alberta, University of Calgary, Calgary, Alberta, Canada; ^2^Department of Radiology, Cumming School of Medicine, Stephenson Cardiac Imaging Centre, Libin Cardiovascular Institute of Alberta, University of Calgary, Calgary, Alberta, Canada

^3^Department of Civil Engineering, Libin Cardiovascular Institute of Alberta and Centre for Bioengineering Research and Education, University of Calgary, Calgary, Alberta, Canada;

***Corresponding Author:**

Paul W.M. Fedak, MD PhD FRCSC FAHA

Professor, Section of Cardiac Surgery, Cumming School of Medicine, University of Calgary

C880, 1403-29 Street NW, Calgary, Alberta, Canada, T2N 2T9

[paul.fedak@gmail.com](mailto:paul.fedak@gmail.com)

**SUPPLEMENTARY FIGURES AND TABLES**

**Figure S1**

**Biochemical and Biomechanical Characterization of Bioscaffolds**

(A) FGF-2 concentration was used to determine the extent of bioactive protein removal from bioscaffolds after guanidine hydrochloride neutralization treatment (N=3/group). We assessed statistical significance by Student’s t-test. Values are reported as mean ± SD. (B) Biomaterial stiffness (elastic modulus) was measured using biaxial mechanical testing in the horizontal and vertical directions of the scaffold materials (N=9/group). We assessed statistical significance by two-way ANOVA. P values obtained were >0.05 for all biomechanical comparisons (group or stretch direction). Box plots represent median, and interquartile range. Whiskers represent maximum and minimum values.

**Figure S2**

**Viability Assessment of Cardiac Fibroblasts on Bioscaffolds**

Representative plots from flow cytometric analysis to determine cell viability. We compared cell viability between human atrial cardiac fibroblasts on cell culture plastic (control), neutralized scaffolds and intact scaffolds (N=3/group). We counted the proportion of cells positive for annexin V to reflect apoptosis and cells positive for propidium iodide (PI) to reflect necrosis. We determined the proportion of live cells (Q3: Annexin V-, PI+), early apoptotic cells (Q1: Annexin V+, PI-), late apoptotic cells (Q2: Annexin V+, PI+), and necrotic cells without apoptosis (Q4: Annexin V-, PI+) by quadrant gating. Viability was not significantly different between groups. We assessed statistical significance using Kruskal-Wallis one-way ANOVA. All values are reported as mean ± SD.

**Figure S3**

**In Vitro Human Cell 3-D Collagen Matrix Bioscaffold Model**

Our in vitro bioassay allows for cell-mediated ECM remodeling to be directly observed and objectively quantified. (A) Intact or chemically neutralized 7x10-cm biomaterial sheets are cut into circular bioscaffolds with a diameter of 10-mm. (B) 1.5-mm diameter defects are created within bioscaffolds and 1 mg/mL collagen solution is polymerized as a microgel collagen matrix into each defect. After collagen is polymerized, human cardiac fibroblasts are seeded at low density to ensure the smallest number of cells per opening. (C) The cell-matrix-bioscaffold constructs are fixed after 24-hours and imaged using confocal reflectance microscopy. Representative confocal images of openings are shown without cells and with cells (red = reflectance collagen fibrils, green = actin). Scale bar = 20 µm.

**Figure S4**

**Intact Bioscaffolds Induce a Pro-Vasculogenic Response from Ventricular Cardiac Fibroblasts**

We show protein concentration of FGF-2 (A), HGF (B), and VEGF (C) as assessed by multiplex analysis of conditioned media from ventricular human cardiac fibroblasts on tissue culture plastic (control), neutralized scaffolds, and intact scaffolds (N=3/group). Values are reported as mean ± SD. We calculated statistical significance using Kruskal-Wallis one-way analysis of variance (ANOVA).

**Figure S5**

**Cardiac Fibroblasts After Injection In Vivo**

Representative images of myocardium with injected rodent cardiac fibroblasts in no scaffold, neutralized scaffold, and intact scaffold treated animals 7 days post-MI. We labeled cardiac fibroblasts isolated from healthy rat hearts with PKH26 (yellow) and injected cells into the myocardium immediately after induction of MI prior to bioscaffold implantation. Arrows indicate injected fibroblasts.

**Table S1**

**Patient Demographics**

Demographic and intraoperative data for the complete cohort of 8 patients. All patients were male with a mean age of 61 ± 8 years. STEMI = ST-Elevation Myocardial Infarction, NSTEMI = Non-ST-Elevation Myocardial Infarction, Y = Yes, N = No.

| Patient # | Sex | Age | Injury Site | Preoperative  Microvascular Obstruction | Time Between Initial Injury and Surgery | Number of Bypasses | Site(s) of Revascularization | Site of Bioscaffold Implantation |
| --- | --- | --- | --- | --- | --- | --- | --- | --- |
| 1 | M | 74 | Inferior  STEMI | Y | 10 days | 2 | Anterior,  Inferior | Inferior |
| 2 | M | 47 | Inferolateral  NSTEMI | Y | 24 days | 2 | Anterior,  Lateral | Inferior |
| 3 | M | 64 | Anterior  STEMI | N | 13 days | 3 | Anterior,  Inferior | Anterior |
| 4 | M | 59 | Anterior  STEMI | N | 9 days | 2 | Anterior | Anterior |
| 5 | M | 57 | Inferolateral  NSTEMI | N | 8 days | 3 | Anterior,  Inferior | Inferolateral |
| 6 | M | 58 | Anterolateral  NSTEMI | N | 17 days | 2 | Anterior,  Lateral | Anterolateral |
| 7 | M | 64 | Inferior  NSTEMI | N | 16 days | 4 | Anterior,  Lateral,  Inferior | Inferior |
| 8 | M | 68 | Inferolateral  NSTEMI | N | 2 days | 3 | Anterior,  Lateral,  Inferior | Inferolateral |

**Table S2**

**Assessment of Cardiac Structure and Function by CMR**

Data are represented as number and percentage. EF = ejection fraction, LVESVI = left ventricular end systolic volume indexed, LVEDVI = left ventricular end diastolic volume.

|  | EF (%) | | | LVESVI (mL/m^2^) | | | LVEDVI (mL/m^2^) | | | | Total Scar (g) | | | |
| --- | --- | --- | --- | --- | --- | --- | --- | --- | --- | --- | --- | --- | --- | --- |
| Patient # | *Baseline* | *6* *Weeks* | *6 Months* | *Baseline* | *6 Weeks* | *6 Months* | *Baseline* | *6 Weeks* | *6 Months* | *Baseline* | | *6 Weeks* | *6 Months* |  |
| 1 | 51 | 50 | 58 | 34 | 35 | 25 | 70 | 70 | 60 | 24 | | 12 | 11 |  |
| 2 | 31 | 36 | 41 | 64 | 55 | 51 | 93 | 86 | 87 | 30 | | 20 | 20 |  |
| 3 | 60 | 63 | 55 | 29 | 31 | 38 | 74 | 84 | 84 | <1 | | <1 | <1 |  |
| 4 | 59 | 57 | 64 | 34 | 36 | 32 | 83 | 83 | 89 | <1 | | <1 | <1 |  |
| 5 | 57 | 57 | 63 | 42 | 35 | 32 | 96 | 82 | 86 | 12 | | 5 | 4 |  |
| 6 | 56 | 62 | 55 | 27 | 19 | 31 | 63 | 49 | 68 | <1 | | <1 | <1 |  |
| 7 | 59 | 63 | 55 | 30 | 28 | 42 | 72 | 74 | 92 | 2 | | <1 | <1 |  |
| 8 | 65 | 55 | 54 | 22 | 27 | 32 | 65 | 60 | 70 | 5 | | 4 | 2 |  |

**Table S3**

**Regional and Global Myocardial Perfusion Change Over Time**

Data are presented as perfusion rate (mL/min/g) for two patients at baseline, 6 weeks post-op and 6-months post-op. The perfusion data is reported for each segment of the left ventricle as per the AHA 17-segment model. Global left ventricular perfusion is also reported.

|  |  | **1** | **2** | **3** | **4** | **5** | **6** | **7** | **8** | **9** | **10** | **11** | **12** | **13** | **14** | **15** | **16** | **Global** |
| --- | --- | --- | --- | --- | --- | --- | --- | --- | --- | --- | --- | --- | --- | --- | --- | --- | --- | --- |
| **Patient 1** | Baseline | 0.54 | 0.83 | 0.55 | 0.47 | 0.42 | 0.39 | 0.41 | 0.47 | 0.54 | 0.83 | 0.56 | 0.39 | 0.41 | 0.56 | 0.74 | 0.40 | 0.53 |
|  | 6 Weeks | 1.31 | 1.34 | 1.06 | 0.43 | 0.76 | 1.05 | 1.31 | 1.34 | 1.06 | 0.43 | 0.76 | 1.05 | 1.29 | 1.28 | 0.46 | 0.91 | 0.99 |
|  | 6 Months | 1.06 | 1.49 | 1.32 | 0.74 | 1.34 | 1.49 | 1.06 | 1.49 | 1.32 | 0.74 | 1.34 | 1.49 | 1.19 | 1.45 | 0.81 | 1.51 | 1.24 |
| **Patient 2** | Baseline | 0.94 | 1.01 | 0.98 | 0.55 | 0.41 | 0.77 | 0.94 | 1.01 | 0.98 | 0.55 | 0.41 | 0.77 | 0.91 | 1.00 | 0.58 | 0.6 | 0.78 |
|  | 6 Weeks | 1.66 | 1.44 | 1.33 | 0.79 | 1.04 | 1.32 | 1.61 | 1.44 | 1.33 | 0.79 | 1.04 | 1.32 | 1.59 | 1.42 | 0.83 | 1.18 | 1.26 |
|  | 6 Months | 1.39 | 1.54 | 1.31 | 0.76 | 1.10 | 1.36 | 1.51 | 1.43 | 1.31 | 0.86 | 1.24 | 1.36 | 1.54 | 1.91 | 1.15 | 1.54 | 1.33 |

**Table S4**

**Gene Expression of Human Cardiac Fibroblasts on Intact Scaffolds**

List of differentially regulated genes from human cardiac fibroblasts seeded on intact scaffolds versus neutralized scaffolds organized by function

| Function | P-Value | Z-Score | Molecules |
| --- | --- | --- | --- |
| Formation of Blood Vessel | 7.8E-09 | 2.024 | *AGT,ANG,ANPEP,APOE,CCL11,CDH5,CDKN2B,CXCL12,*  *EFNB1,EFNB2,F3,FGF2,IL1B,IL33,INPP4B,ITGB3,SERPINF,*  *SPARC,SRC,SUFU,TNFSF10,TUBA4A,VASH1,VEGFA* |
| Cellular Homeostasis | 3.9E-09 | 2.028 | *A2M,ACACB,AGT,ALOX15,AMPD3,ANGPTL4,ANXA1,APOE,AQP1,AQP3,ATP1A1,BAG3,BDKRB1,BDKRB2,C3AR1,C7,C9orf72,CADPS2,CCDC3,CCL11,CDH5,COL4A1,CTSL,CX3CL1,CXCL1,CXCL12,CXCL2,CXCL3,CXCL8,CYLD,CYP19A1,DCN,DMD,DUSP4,EFNB1,EFNB2,ENG,F3,FGF2,FZD2,GAA,GCKR,GHR,GK,GSN,HMGA1,HSPB8,IFNE,IL11RA,IL13RA2,IL1A,IL1B,IL1RN,IL24,IL33,IL36B,INPP4B,INPP5D,ITGB3,KCNJ2,KDM4A,LFNG,LIF,LZTS1,MAFG,MAP1LC3B,MAPK13,MET,MMP1,MMP10,MOAP1,MYADM,NAMPT,NDRG1,NFIL3,NPC1,NQO1,PENK,PID1,PIK3CB,PLCE1,POPDC3,PRKCA,PRNP,PTGER4,PTPN22,RAC2,RNF152,RUBCN,SERPINA3,SFRP4,SLC12A4,SLC14A1,SLC3A2,SMYD3,SNAI2,SOAT1,SOCS5,SQSTM1,SRC,SRXN1,STC1,TCF4,TCF7,TIMP3,TMEM38B,TNFRSF11A,TNFSF10,TP53BP2,TRIB2,TSLP,UBQLN1,UBR4,VEGFA,VPS37A,XPO1,ZMAT3* |
| Chemotaxis | 3.7E-08 | 2.129 | *A2M,ADGRA2,AGT,ANOS1,ANXA1,APOE,BDKRB2,BMP2,C3AR1,CCL11,CX3CL1,CXCL1,CXCL12,CXCL2,CXCL3,CXCL8,CYP19A1,EFNB1,EFNB2,ENG,FGF2,FLOT1,FOSL1,GSN,IL1A,IL1B,IL33,INPP5D,ITGA2,ITGB3,LIF,MET,NFKB2,PENK,PIK3CB,PRKCA,PRNP,PTGES,RAC2,RARRES2,RGS3,S100A4,SERPINA3,SERPINF1,SLIT3,SNAI2,SRC,TNFRSF6B,TSLP,TXN,VEGFA* |
| Proliferation | 6.3E-11 | 2.238 | *AEBP1,AGT,AQP1,ATF3,BMP2,CCDC80,CCND2,CDKN2B,CDKN2C,COL14A1,CXCL3,DCN,DKK3,DUSP1,DUSP4,ENG,FGF2,FOSL1,IL1B,IL24,ITGB3,LIF,LUM,LZTS1,MET,NDRG1,PLCE1,PML,POSTN,PPM1D,PTGES,SERPINF1,SLC7A11,SPARC,SRC,SULF1,SULF2,TGIF1,TMPO,TNFSF10,TXN,VCAN* |
| Cell Viability | 6.2E-09 | 2.735 | *ADK,AGRN,AGTRAP,APOE,AQP3,ATF3,BAG3,BMP2,CAMK2N1,CCL11,CDH5,COL1A1,CX3CL1,CXCL1,CXCL12,CXCL2,CXCL3,CXCL8,CYLD,DUSP1,DYRK3,EFNB1,ENG,EPHB3,ETV6,FES,FGF2,FOSL1,FZD2,GCH1,GCLC,GHR,HMGA1,HSPB8,IL11RA,IL1A,IL1B,IL1RN,IL24,IL33,INPP5D,LAMA5,LIF,MAP1LC3B,MAPK10,MET,MMP1,NAMPT,NCAM1,NDRG1,NFIL3,NFKB2,NPC1,NT5C3A,PCDHGB7,PCNA,PENK,PGRMC1,PIK3CB,PLCE1,PML,POLH,POR,POSTN,PPM1D,PRKCA,PRNP,PTGIS,PTGR1,PTPN22,RAC2,RBP1,S100A4,SERPINB2,SERPINF1,SNAI2,SOX9,SPARC,SQSTM1,SRC,SSPN,STC1,STX1A,SUFU,SULF1,TCF7,TDP2,TFPI,TIMP3,TMEM158,TNFSF10,TP53BP2,TP53I3,TRIB2,TSLP,TXN,TYMS,UBQLN1,VCAN,VEGFA,XAF1,XPO1* |
| Degradation of Connective Tissue | 2.3E-10 | 2.141 | *A2M,BDKRB1,CXCL12,CXCL8,FGF2,HTRA1,IL1A,IL1B,IL1RN,IL33,MMP1,MMP10,MMP3,NAMPT,NFIX,SPARC,SULF1,SULF2,TIMP3* |
| Cell Death | 5.1E-07 | -2.05 | *ALOX15,BIRC3,CDCP1,CDKN2C,CFH,COL4A1,DUSP1,FGF2,G6PD,HMGA1,IL36RN,MET,MOAP1,NQO1,PML,POLH,PPM1D,PRKCA,RHOBTB2,S100A4,SERPINA3,SERPINF1,SLC7A11,SRC,TNFSF10,TRIAP1,UACA,VEGFA,XPO1* |
| Inflammatory Response | 1.6E-06 | -2.034 | *ADK,AGT,ALOX15,AMPD3,ANXA1,APOE,AQP1,ATF3,BIRC3,C3AR1,CCL11,CD14,CDH11,CFH,COL1A1,COL1A2,COL3A1,COL4A1,CTSL,CX3CL1,CXCL1,CXCL12,CXCL2,CXCL3,CXCL8,CYLD,CYP19A1,DCN,DUSP1,F3,FAH,GAA,GPX7,HPRT1,IL11RA,IL13RA2,IL1A,IL1B,IL1RN,IL24,IL33,INPP5D,ITGB3,KLF3,LIF,MAP1LC3B,MME,MMP10,NFIL3,NFKB2,NOD1,NPC1,NQO1,PLA2R1,PLCE1,POR,PTGES,PTPN22,S100A4,SLC7A11,SOAT1,SPARC,SQSTM1,SRC,ST3GAL3,TCF4,TFPI,TIMP3,TNFSF10,TSLP,TYMS,UACA,VEGFA* |
